# Analysis of Metagenomics Next Generation Sequence Data for Fungal ITS Barcoding: Do You Need Advance Bioinformatics Experience?

**DOI:** 10.3389/fmicb.2016.01061

**Published:** 2016-07-26

**Authors:** Abdalla Ahmed

**Affiliations:** College of Medicine, Umm Al-Qura UniversityMakkah, Saudi Arabia

**Keywords:** ITS sequencing, next generation sequencing, fungi, bioinformatic tools and databases, metagenomics

## Abstract

During the last few decades, most of microbiology laboratories have become familiar in analyzing Sanger sequence data for ITS barcoding. However, with the availability of next-generation sequencing platforms in many centers, it has become important for medical mycologists to know how to make sense of the massive sequence data generated by these new sequencing technologies. In many reference laboratories, the analysis of such data is not a big deal, since suitable IT infrastructure and well-trained bioinformatics scientists are always available. However, in small research laboratories and clinical microbiology laboratories the availability of such resources are always lacking. In this report, simple and user-friendly bioinformatics work-flow is suggested for fast and reproducible ITS barcoding of fungi.

## Introduction

Since the introduction of Sanger Sequencing, many microbiology laboratories started using DNA sequence data for microbial identification and genotyping. These DNA sequence data revolutionized microbial genotyping and taxonomy and quickly became part of the routine clinical microbiology work (Makimura, 2001; Leaw et al., 2006). DNA sequence data generated by Sanger Sequencing technology characterized by relatively limited size (±800 bases single read) and high base calling quality. This nature of Sanger sequence data enable most scientists, with no standard bioinformatics training, to perform many basic sequence data analysis without the need of highly trained bioinformatics specialists. However, with the introduction of next-generation sequencing (NGS) technologies, huge sequence data with varying degrees of quality become available. The analysis of such large and complex data become rather difficult. Therefore, it become mandatory, for many research centers, to recruit specially trained bioinformatics staff to handle the huge NGS data obtained from these diverse sequencing platforms. Alternatively, many small research centers and microbiology laboratories are forced to seek help in data analysis from specialized sequencing centers or bioinformatics commercial services providers.

In recent years, and with the great development of NGS platforms and sequencing technologies, DNA sequencing in no longer done in specialized sequencing centers and reference research laboratories only. Library preparation protocols for NGS become simple and acquisition of next generation sequencers become affordable by many research and diagnostics laboratories. Therefore, it become important for microbiologists to know how to make sense of the massive NGS data generated by these new sequencing technologies. In reference laboratories, the analysis of such data is not a big deal, since suitable IT infrastructure and well-trained bioinformaticians are always available. However, in small research laboratories and clinical microbiology laboratories the availability of such resources are always lacking.

Microbial DNA sequencing applications are numerous and these applications are rapidly evolving with introduction new sequencing technologies. In clinical microbiology, NGS data can be used in many routine applications. For example, whole microbial genome sequencing and targeted sequencing are currently widely used for unlimited applications such as species identification, virulence genes detection, antimicrobial resistant mechanisms prediction and genotyping (Zankari et al., 2012; Joensen et al., 2014; Larsen et al., 2014; Garnaud et al., 2015). Another interesting area for microbiologist is metagenomics, which can be used for sequencing of novel species from environmental specimens. Metagenomics can also be used for species identification of bacteria and fungi by targeted sequencing of the 16S and ITS regions of the rRNA genes, respectively (Salipante et al., 2013; Tang et al., 2015).

## Why Do We Need to Sequence DNA for Species Identification?

Species identification in fungi is difficult and time-consuming even for those with special training and experience in medical mycology. Therefore, it becomes routine in many centers to sequence the Internal Transcribed Spacer (ITS) region of the ribosomal RNA genes (rDNA) for species identification. The rDNA of fungi exist as a multiple-copy gene family comprised of highly similar DNA sequences (typically from 8 to 12 kb each). The ITS region of the rDNA is the most widely sequenced DNA region in fungi. ITS is typically most useful for molecular systematics at the species level, and even within species. This is because ITS characterized by high degree of variation than other regions of rDNA such as small sub unit (SSU) and large sub unit (LSU) of the rDNA.

## Why Do We Need Help in “Bioinformatics” But Not in “DNA Sequencing”?

Sequencing library preparation workflow is getting much easier. Thanks for the innovative, simple and quick library preparation protocols for DNA sequencing. However, data analysis remains the most challenging step in this wonderful technology. NGS data analysis is the biggest challenge in routine application of NGS in clinical setting (Desai and Jere, 2012). NGS data analysis is rapidly evolving field, but still largely carried out using commercial and/or open source research tools not designed for clinical laboratories (Desai and Jere, 2012). One another issue on NGS data analysis is the huge amount of data generated, which is beyond the computing infrastructure of most clinical setting (Stein, 2010). In fact, most of NGS platforms have some data analysis functionality, which can be done on the same sequencing machine. However, these automated bioinformatics workflows does not provide total analysis solutions, and it remains difficult and unclear for many microbiologist how to quickly and efficiently analyze the raw sequencing data to get clear answers for many basic questions.

In this report, we present a simple and easy bioinformatics workflow for one of the commonly asked questions, “what is the species of this fungal isolate”? The workflow consist of sequences quality check, *de novo* assembly and sequence similarity search. The workflow is based on two genomics computing environments Illumina BaseSpace^1^ and the Public Galaxy Server (Galaxy Project^2^). This bioinformatics workflow needs only basic bioinformatics knowledge, and can be done by any scientist using any computer connected to the internet.

## Simple Data Analysis Workflow:

Regardless of NGS platform used, sequence data normally stored in text file in a Fastq format, which contains sequence data and the quality score of base calling for each base. This Fastq file is your starting material. If you are using paired end library you will end with two Fastq files one for each read (read 1 and read 2). Before start analyzing the data, raw sequence reads need to checked for quality. The most important quality parameters are quality score of base calling, number of reads and reads length distribution. In addition, sequence reads need to be checked for possible sequencing adapter contamination, especially if using small sequencing target. Low quality sequence reads and/or sequence contamination need to be removed from data sets before any subsequent data manipulation or analysis. For quality check of sequencing read, we recommend the use of FastQC tool, which is available as a push-button tool at the Public Galaxy Server^2^) and Illumina BaseSpce (BaseSpace Labs, Illumina, San Diego, CA, USA).

Once sequence data has been checked for quality, the next step is to assemble the sequence reads into contigs using any short sequence *de novo* assembler. The aim of this *de novo* assembly is to covert the large number of reads into few contigs (a set of overlapping DNA segments that together represent a consensus region of DNA). Assembled contigs can be easily used for sequence similarity search and species identification. In this workflow, we recommend the use of Velvet assembler or SPAdes Genome Assembler 3.0 for the *de novo* assembly. These two applications are in the Illumina BaseSpace applications^1^. Sequence reads can also be assembled using many other free or commercial tools. Once assembly is finished, assembled contigs can visualized using any text viewer such as Notepad or the Universal Viewer^3^. The best contigs with sizes matching the expected sequences regions can be directly used for ITS based species identification at the NCBI Nucleotide BLAST^4^ or the ISHAM ITS database^5^ (Irinyi et al., 2015).

## Conclusion

Analysis of NGS data for ITS-based fungal identification is easy to perform and does not require advance bioinformatics training or expensive IT infrastructure. However, in addition to the available bioinformatics tools, there is a need for more automated data interpretation tools, which are able to generate easily understandable clinical reports. When such tools become available, NGS-based identification and other microbial sequence-based applications will be part of the clinical microbiology routine work.

## Author Contributions

The author confirms being the sole contributor of this work and approved it for publication.

## Conflict of Interest Statement

The author declares that the research was conducted in the absence of any commercial or financial relationships that could be construed as a potential conflict of interest.

## Footnotes

1 https://basespace.illumina.com

2 https://usegalaxy.org

3 http://www.uvviewsoft.com/uviewer/

4 http://blast.ncbi.nlm.nih.gov/Blast.cgi

5 http://its.mycologylab.org/Biolomicssequences.aspx
